# Sputum Biomarkers and the Prediction of Clinical Outcomes in Patients with Cystic Fibrosis

**DOI:** 10.1371/journal.pone.0042748

**Published:** 2012-08-10

**Authors:** Theodore G. Liou, Frederick R. Adler, Ruth H. Keogh, Yanping Li, Judy L. Jensen, William Walsh, Kristyn Packer, Teresa Clark, Holly Carveth, Jun Chen, Shaunessy L. Rogers, Christen Lane, James Moore, Anne Sturrock, Robert Paine, David R. Cox, John R. Hoidal

**Affiliations:** 1 Department of Medicine, School of Medicine, University of Utah, Salt Lake City, Utah, United States of America; 2 Division of Respiratory, Critical Care and Occupational Pulmonary Medicine, University of Utah, Salt Lake City, Utah, United States of America; 3 Intermountain Adult CF Center, University of Utah, Salt Lake City, Utah, United States of America; 4 Department of Mathematics, University of Utah, Salt Lake City, Utah, United States of America; 5 Department of Biology, University of Utah, Salt Lake City, Utah, United States of America; 6 Department of Medical Statistics, London School of Hygiene and Tropical Medicine, London, United Kingdom; 7 Department of Statistics and Nuffield College, Oxford, United Kingdom; Abramson Research Center, United States of America

## Abstract

Lung function, acute pulmonary exacerbations (APE), and weight are the best clinical predictors of survival in cystic fibrosis (CF); however, underlying mechanisms are incompletely understood. Biomarkers of current disease state predictive of future outcomes might identify mechanisms and provide treatment targets, trial endpoints and objective clinical monitoring tools. Such CF-specific biomarkers have previously been elusive. Using observational and validation cohorts comprising 97 non-transplanted consecutively-recruited adult CF patients at the Intermountain Adult CF Center, University of Utah, we identified biomarkers informative of current disease and predictive of future clinical outcomes. Patients represented the majority of sputum producers. They were recruited March 2004-April 2007 and followed through May 2011. Sputum biomarker concentrations were measured and clinical outcomes meticulously recorded for a median 5.9 (interquartile range 5.0 to 6.6) years to study associations between biomarkers and future APE and time-to-lung transplantation or death. After multivariate modeling, only high mobility group box-1 protein (HMGB-1, mean = 5.84 [log ng/ml], standard deviation [SD] = 1.75) predicted time-to-first APE (hazard ratio [HR] per log-unit HMGB-1 = 1.56, *p-*value* = *0.005), number of future APE within 5 years (0.338 APE per log-unit HMGB-1, *p*<0.001 by quasi-Poisson regression) and time-to-lung transplantation or death (HR = 1.59, *p* = 0.02). At APE onset, sputum granulocyte macrophage colony stimulating factor (GM-CSF, mean 4.8 [log pg/ml], SD = 1.26) was significantly associated with APE-associated declines in lung function (−10.8 FEV_1_% points per log-unit GM-CSF, *p*<0.001 by linear regression). Evaluation of validation cohorts produced similar results that passed tests of mutual consistency. In CF sputum, high HMGB-1 predicts incidence and recurrence of APE and survival, plausibly because it mediates long-term airway inflammation. High APE-associated GM-CSF identifies patients with large acute declines in FEV_1_%, possibly providing a laboratory-based objective decision-support tool for determination of an APE diagnosis. These biomarkers are potential CF reporting tools and treatment targets for slowing long-term progression and reducing short-term severity.

## Introduction

Cystic fibrosis (CF) is the most common lethal genetic disorder among Caucasians, but disease occurs world-wide. Approximately 10 million Americans carry mutations while 25,000 suffer actual disease [1]. Pancreatic enzyme therapy increased survival from infancy to 6 years of age and shifted the commonest cause of death from starvation to lung disease, which remains life-limiting for most patients [1], [2]. Median predicted survival now approaches 40 years [1] but remains well short of normal.

Therapeutic successes have greatly increased CF survival, however, this makes survivorship problematic for assessing treatment effects, prompting a search for alternative measurements [2]–[4]. Lung function, acute pulmonary exacerbations (APE) of CF requiring hospitalization and weight best predict survivorship clinically [2] but may themselves require large trial enrollments and years of observation to discern treatment effects.

In CF, airway inflammation underlies pulmonary deterioration, frequent APE and persistent malnutrition [5], [6]. Chronic airway infections incite intense, dysfunctional inflammation [5], [7], eliciting multiple deleterious signals [6]. However, these inflammatory signals may serve as immediate biomarkers of disease state. A biomarker that additionally predicts clinical outcomes might identify causal mechanisms for airway disease, pinpoint investigational targets, rapidly detect efficacy of new therapies, and distinguish patients most needing urgent interventions [6], [8]. Such biomarkers have previously been elusive in CF.

We hypothesized that high-mobility group box-1 protein (HMGB-1), a novel inflammatory cytokine, might be informative of future clinical events in CF. HMGB-1 is a highly conserved DNA-shepherding protein. However, when released by necrosis or secretion, HMGB-1 acts as a potent cytokine associated with delayed onset of prolonged inflammation [9], [10] and septic shock in animal models reversible with anti-HMGB-1 antibodies [11]. In this work, we investigated the potential of sputum HMGB-1 measurements to reflect concurrent clinical status and predict future outcomes following a physician-patient encounter [8].

## Methods

### Ethics Statement

This study was reviewed and approved by the University of Utah Investigational Review Board prior to enrollment of any patients. All participants were included only after obtaining written informed consent for sputum collection and analysis. Collection of information for the CF Foundation Patient Registry (CFFPR) was reviewed and approved at each participating institution and by the University of Utah Investigational Review Board. Our use of data from the CFFPR was also reviewed and approved by the CF Foundation. All adult patients included in the CFFPR gave written informed consent. All pediatric patients in the CFFPR gave assent if able to understand, and their parents or guardians gave written informed consent and permission to participate. The study was conducted under the supervision of the physicians involved (TGL, WW, HC, RP III, JRH), and patients were advised of risks, benefits and the right to withdraw from further involvement in the study at any point without repercussions. All data, particularly patient identifying data, were physically and electronically secured throughout the study.

### Study Outline

From March, 2004 through May, 2011, we recruited sputum-producing outpatients from the Intermountain Adult CF Center at quarterly-regular and unplanned-sick visits. All visits were conducted in compliance with CF Foundation guidelines for outpatient care [12]. We collected sputum, recorded same-day lung function, weight, height, sputum culture results, number of prior-year APE, and recorded whether patients were in stable, mild exacerbation or APE states. We actively followed patients to lung transplantation, death, loss-to-follow-up, or May 31, 2011, the end of study, recording APE numbers and spirometry results [13]. Patients recruited from March, 2004 through September 2006 formed the study groups while remaining patients formed validation groups (Tables 1 and 2). Forced expiratory volume in 1 second (FEV1) was measured [13] and normalized to percent predicted FEV_1_ (FEV_1_%) [14]. We assessed generalizability of results by comparing our patients with sputum-producing adults in the 2006 CFFPR.

**Table 1:** Study Design^a^.

	Study Group 1	Study Group 2	Study Group 3	Validation Group 1^b^	Validation Group 2^b^	Validation Group 3^b^	Validation Group 4^b^
N	56	26	76	27	17	21	9
Measures	All biomarkers	All biomarkers in pairs	HMGB-1	HMGB-1	HMGB-1	GM-CSF	GM-CSF
Analyses Applied	1	2, 3, 4	5	4	4, 5^c^	3	3

a A total of 97 unique patients participated in the study including study and validation groups.

b Validation Groups had no overlapping patients with Study Groups that underwent the same analysis with one exception (please see the next footnote). For Analysis 3, for example, no patients were found in both Study Group 2 and Validation Groups 3 and 4.

c Eight of the 17 patients in Validation Group 2 were excluded from analysis 5 because they were already included with Study Group 3 leaving 9 patients for the validation and mutual consistency testing (see also text and Table 7).

**Table 2:** Analysis Descriptions.

Analysis	Statistical Techniques	Relationships Explored (Results Location)
1^a^	Linear Regression	Biomarkers with concurrent FEV_1_% and weight-for-age z-score (Table 5, Table S2)
	Quasi-Poisson Regression	Biomarkers with number of APE in year prior to sputum sample collections (Table 5, Table S2)
	Logistic Regression	Biomarkers with *Pseudomonas aeruginosa* and *Staphylococcus aureus* infections (Table S2)
2	Linear Regression	Biomarker changes between stable and APE states (Figure S1D-F, Table S1)
3	Linear and Quasi-Poisson Regressions	Biomarkers from stable or APE states or the change in biomarkers between stable and APE states with clinical outcomes such as APE-associated decline in FEV_1_% and numbers of future APE. (Figure 1C–D, Table 5, Table S3)
4	Proportional Hazards Modeling	Biomarkers and time-to-first APE (Figure 2A, Table 6, Figure S2)
5	Proportional Hazards Modeling	Biomarkers and time-to-lung transplantation or death (Figure 2B, Table 6)
Validations	Linear and Quasi-Poisson Regressions, Proportional Hazards Modeling, Mutual Consistency Testing	HMGB-1 and GM-CSF levels and Clinical Predictions (Table 7)

a We examined between-biomarker correlations to help interpret results of multivariate models involving multiple potential biomarkers (Table S4).

### Acute Pulmonary Exacerbations

We diagnosed APE and hospitalized for symptoms and objective evidence of severe acute worsening of CF. Symptoms included increased cough, sputum production or dyspnea, chest pain or tightness, hemoptysis, fever, chills, arthralgias or decreased exercise tolerance. Patients had to meet one objective criterion: 10% decrease in FEV_1_% or percent predicted forced vital capacity, fever above 38.4°C, documented hemoptysis greater than 100 ml per episode, SaO_2_ below 90% or PaO_2_ below 60 mm Hg despite usual oxygen, increased supplemental oxygen requirements or three or more kg unplanned drop in weight within 3 months. Symptomatic patients without objective findings were classified as mild exacerbation and not hospitalized. These criteria were modified from previous APE definitions to allow prospective application at a patient encounter by excluding retrospective criteria such as prior antibiotic treatment [2], [4], [15].

### Sputum Evaluations

Sputum samples underwent standardized processing (see Text S1). CF Foundation guidelines were followed to collect sputum cultures. Cultures were obtained within 6 months of sputum collections as bacterial infections are typically stable over such a period [16], a finding that has recently been supported by culture-independent methods [17]. We measured potential biomarkers previously identified as important in CF including granulocyte macrophage colony stimulating factor (GM-CSF) [6], [8], [18]–[25] using SearchLight multiplex assay services (Aushon Biosystems, Billerica, MA). We measured HMGB-1 by ELISA using commercially-available antibodies (R&D Systems, Inc, Minneapolis, MN) and previously published protocols [26] after confirming assay reproducibility on consecutive days (see Text S1). Biomarker measurements reported are log-scale.

### Statistical Analysis

Through 5 analyses [27], we examined (1) associations between biomarkers and concurrent clinical disease measurements, (2) APE effects on biomarkers, (3) biomarker predictions of clinical outcomes using linear, logistic and quasi-Poisson regression [28], and proportional hazards modeling [29], [30] (See Tables 1 and 2 and Text S1 for details). Throughout this paper, *p*-values smaller than 0.05 are reported as statistically significant. In Analyses 1–3, we performed multiple univariate tests for associations between biomarker values and various clinical measurements, and we used stringent Bonferroni correction by dividing 0.05 by the number of tests performed to define the corrected *p*-value required for significance. We repeated Analyses 3–5 using sputum measurements from 4 sets of validation patients chosen so that no patient appeared simultaneously in a study group and comparison validation groups (Table 1). Mutual consistency of results was assessed by a weighted least squares analysis.

## Results

### Patients and Samples

During enrollment of the three study groups, 161 patients attended clinic at least once; 26 produced no sputum. Including validation groups, we recruited 97 patients and collected 149 samples: 56 samples were collected during stable, 31 during mild exacerbation and 62 during APE clinical states (Table 3 and Text S1). Every sputum-producing patient approached agreed to participate, but we were occasionally limited by availability of study personnel or by temporary lack of sputum production. All patients were lifetime non-smokers. Study patients were nearly indistinguishable from adult sputum producers in the 2006 CF Foundation Patient Registry (Table 4). Our patients had a weight-for-age *z*-score 0.24 units less than the national median and used oral steroids less frequently.

**Table 3:** Patient Characteristics^a^.

	All patients	Study Group 1	Study Group 2^b^	Study Group 3	Validation Group 1	Validation Group 2	Validation Group 3	Validation Group 4
N	97	56	26	76	27	17	21	13
Gender, Percent Male	54	55	58	57	59	41	52	46
Age, years	24.9 (21.9–30.3)	25.3 (22.2–29.6)	24.8 (21.1–31.3)	25.6 (21.8–30.7)	25.4 (21.9–29.6)	23.1 (21.4–30.2)	27.9 (24.2–36.2)	22.7 (21.5–24.1)
FEV_1_%	59 (43–75)	59 (34–71)	46 (33–54)^c^	56 (40–71)	71 (57–84)	45 (30–59)	68 (44–83)	60 (45–80)
Prior Year APE	1 (0–2)	1 (0–1)	1 (1–2)	1 (0–1)	1 (0–1)	1 (0–2)	1 (0–1)	1 (0–3)
Weight, kg	57.5 (51.4–65.4)	58.1 (51.4–66.1)	57.2 (47.4–60.9)	58.2 (52.3–66.1)	59.7 (55.4–72)	53.6 (50.9–59.9)	58 (53.1–63.6)	54.6 (48.6–68.6)
Height, cm	168 (161–176)	169 (162–176)	168 (162–173)	169 (160–176)	169 (162–179)	168 (162–176)	169 (156–174)	167 (158–176)
Body Mass Index	20.5 (19.1–22.1)	20.6 (19.2–22.0)	20.4 (18.0–21.8)	21.0 (19.2–22.1)	21.3 (20.1–22.4)	20.0 (18.9–20.5)	21.0 (19.6–22.0)	20.3 (18.5–21.1)
Patients with CF-Related Diabetes, percent	22	14	58^c^	22	0	18	9.5	31
Percent likelihood of surviving 5 years, median (interquartile range)^e^	0.91 (0.75–0.98)	0.93 (0.76–0.98)	0.77 (0.63–0.90)^c^	0.92 (0.75–0.97)	0.97 (0.90–0.98)	0.81 (0.76–0.94)	0.94 (0.85–0.99)	0.87 (0.79–0.98)
Number of Deaths	11	5	4	9	0	2	2	2
Number of Lung Transplants	6	5	1	6	0	3	2	0
Infections, percent infected								
* P aeruginosa*	76	77	88	84	74	59	95	77
* S aureus*	33	30	27	26	30	47	29	31
Neither *S aureus* nor *P aeruginosa*	11	9	12	7	3.7	18	0	15
Both *P aeruginosa* and *S aureus*	21	16	27	17	7.4	24	24	23
Anti-Inflammatory Therapy, percent treated								
Chronic azithromycin	56	50	54	53	59	71	33	77
Inhaled steroids	60	54	62	59	59	65	67	85
Oral steroids	4	4	0	2.6	4	12	5	8
Multiple therapies	42	38	38	50	48	47	33	69

a Results are median (interquartile range) unless noted.

b This group of patients provided two samples each, one from a stable state and one from an APE state at admission for a hospitalization. Data shown here are derived from the time point of the stable sample collection for each individual.

c The 26 patients that gave paired samples necessarily suffered an APE during the study in order to give the necessary APE state sputums. This criterion selected patients with significantly lower lung function, *t*-test *p* = 0.005, increased incidence of CF-related diabetes, χ-square *p*<0.001, decreased 5-year predicted survival, *t*-test *p* = 0.01 and more frequent APE (differences not tested due to confounding) than the other patients in the study.

d Patients in Validation Group 1 had higher FEV_1_% and 5-year predicted survival and remarkably no incidence of CF-related diabetes. Despite these differences, the coefficients for HMGB-1 reported in Table 7 for Validation Groups 1 and 2 are quite similar to those for Study Group 1 patients and pass testing for mutual consistency.

e The 5-year predicted survival is a clinically useful composite estimate of overall disease state in CF but may be difficult to use in interpretation of inflammatory states. Similar to lung function and other clinical markers of disease, it may require years to see a change [2].

Study group patients were followed up to 7.1 years (median 5.9, interquartile range 5.0 to 6.6) through death, lung transplantation, loss to follow-up or end of study. All APE hospitalizations occurred within our center. Nine deaths occurred between 0.6 and 6.5 years, and six lung transplants between 1 and 6.3 years after enrollment. Three patients were lost to follow-up 2.7, 3.4 and 3.6 years after enrollment when they moved.

**Table 4:** Patient Comparisons with the 2006 CF Foundation Patient Registry^a^.

Group	All study patients	2006 CFFPR	*p* b
N	97	7006	–
Gender, Percent Male	54	54	0.93
Age, years	24.9 (21.9–30.3)	26.2 (21.6–34.6)	0.08
FEV_1_%	59.1 (43–75)	58.9 (41.8–77.2)	>0.99
Prior Year APE	1 (0–2)	1 (0–2)	0.29
Weight, kg	57.5 (51.4–65.4)	60 (52.7–69.5)	0.17
Height, cm	168 (161–176)	168 (160–175)	0.7
Weight-for-age z-score	−0.58 (−1.14–0.04)	−0.34 (−1.16–0.30)	0.018
Diabetes, Percent affected	22	24	>0.99
5-Year Predicted Survival	0.91 (0.75–0.98)	0.94 (0.82–0.98)	0.097
**Infections, percent infected**			
* Pseudomonas aeruginosa*	76	80	0.49
* Staphylococcus aureus*	33	39	0.28
Both *P aeruginosa* and *S aureus*	21	28	0.13
Neither *P aeruginosa* nor *S aureus*	11	9.4	0.64
**Anti-Inflammatory Therapy, percent treated**			
Chronic azithromycin	56	57	0.86
Inhaled steroids	60	56	0.48
Oral steroids	4.1	12	0.034
2 or 3 anti-inflammatory agents	42	42	0.94

a Results are median (interquartile range) unless noted. CFFPR patients include all sputum-producing adult patients in 2006 but exclude those followed at the Intermountain Adult CF Center.

b We used χ-square tests to determine statistical differences in Gender, Infections and Anti-inflammatory Therapy between the Intermountain CF Center and the CFFPR 2006. For all other variables shown, we used Kolmogorov-Smirnov tests because data were not normally distributed.

Biomarkers were measured (Table S1) in three overlapping patient study groups after sample inventory demonstrated feasibility of planned analyses (Table 1). Group 1: the initial sputum sample from each of 80 randomly selected patients underwent SearchLight multiplex assays (Aushon); results for 24 were discarded due to unreliability (as samples thawed in transport to Aushon), leaving results from 56 patients for analysis. Group 2: we measured HMGB-1 by ELISA and obtained a second SearchLight multiplex assay (Aushon) on samples from the 26 patients that during repeated collections resulted in a pair of samples per patient: one from an APE and one from a stable point adjacent in time. Ten gave a clinically-stable-time-point sample first; 16 gave an APE-time-point sample first. The median number of days from stable to APE sample was 98 (interquartile range 91–154) and from APE to stable sample was 119 (interquartile range 84–212). Group 3: 76 patients from Group 1 had sufficient sample volumes to measure HMGB-1.

### Analysis 1: Biomarker Measurements and Concurrent Clinical Status

**Figure 1:**
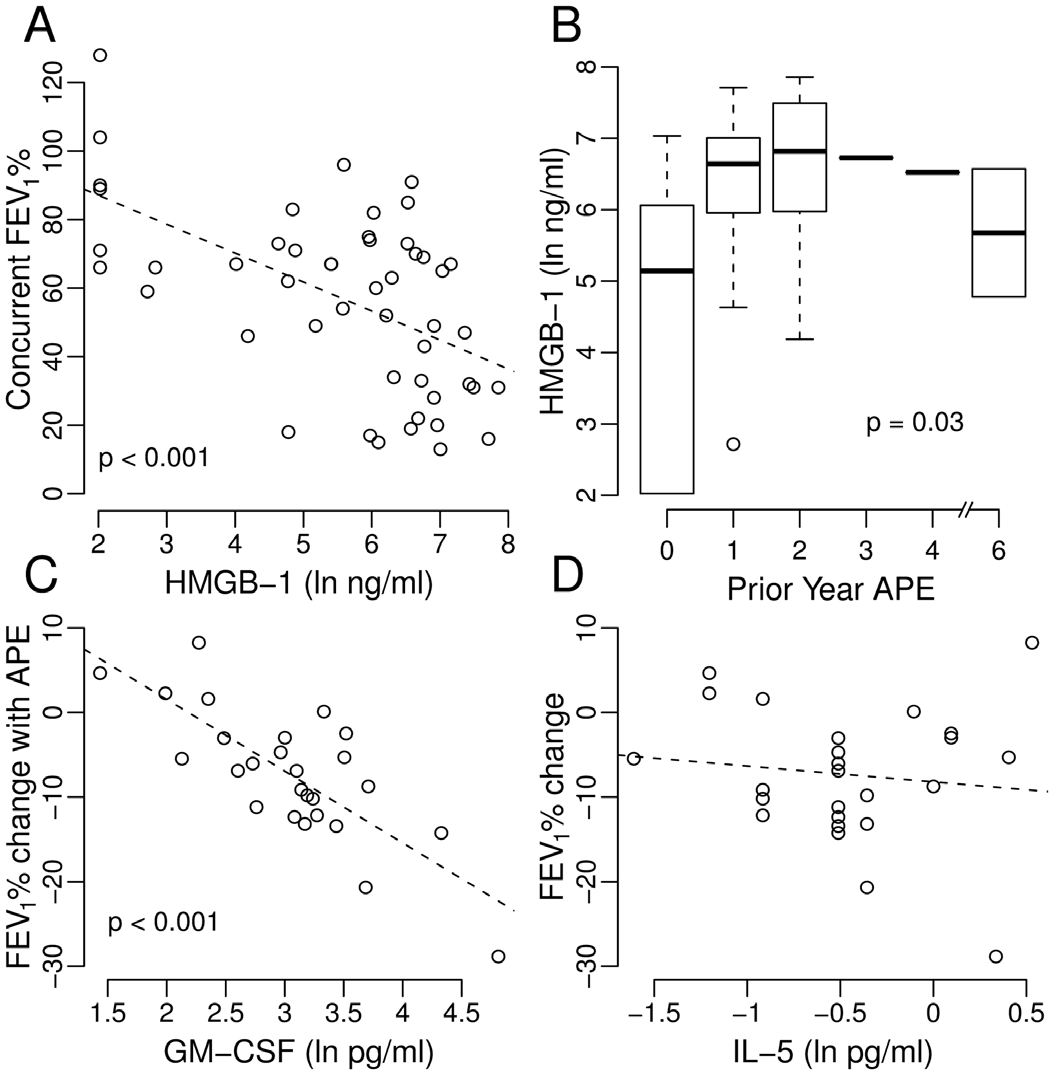
Key Univariate Relationships. HMGB-1 had strong statistically significant associations with A) concurrent FEV1% and B) number of APE suffered in the year prior sputum sample collection. These results illustrate the immediate clinical relevance of HMGB-1. C) GM-CSF measured at APE time-points had an extremely strong univariate association with the size of the APE-associated decline in FEV_1_% for each of 26 patients in Group 2. D) Although the univariate relationship with APE-associated FEV_1_% decline was weak (Table S3), the addition of IL-5 as a covariate to GM-CSF significantly strengthened the multivariate linear regression model of APE-associated decline in FEV_1_% (Table 5).

We looked for associations with concurrent clinical findings to identify plausible biomarkers for CF airway inflammation (Table S2). Backwards model selection using biomarkers with statistically significant or borderline univariate associations (p<0.1) produced three final multivariate models. HMGB-1 and interleukin-(IL)-17A are associated with concurrent FEV_1_% (Figure 1A and S1A), IL-17A with weight-for-age *z*-score (Figure S1B), and HMGB-1 and interferon-(IFN)-α with number of prior-year APE (Figure 1B, Figure S1C, Table 5). (Discussion of these results can be found in Text S1.).

**Table 5:** Multivariate models for concurrent outcomes and APE-associated predictions.

Outcome	Biomarker	Estimates	Standard Error	95% Confidence Interval	*p*-value
**Concurrent Outcomes** a					
FEV_1_%	Intercept	79.1	10.7	–	–
	IL-17	8.41	1.88	4.72 to 12.1	<0.001
	HMGB-1	−4.86	1.76	−8.31 to −1.4	0.008
Weight-for-age *z*-score	Intercept	−0.742	0.111	–	–
	IL-17	0.233	0.0666	0.103 to 0.364	<0.001
Prior-year APE	Intercept	0.47	1.15	–	–
	IFN-α	−1.01	0.355	−1.71 to −0.317	0.006
	HMGB-1	0.288	0.131	0.0314 to 0.544	0.033
**APE-associated Predictions** b					
APE-associated decline in FEV_1_%^c^	Intercept	28.5	5.03	–	–
	GM-CSF (APE)	−10.8	1.44	−13.7 to −8	<0.001
	IL-5 (APE)	6.12	2	2.2 to 10	0.006
Predicted APE during follow up^d^	Intercept	−2	0.797	–	–
	Number of Prior APE	0.739	0.233	0.282 to 1.2	0.004
	Follow Up Time (Years)	0.352	0.0997	0.157 to 0.548	0.002
	HMGB-1 (Stable)	0.338	0.0809	0.18 to 0.497	<0.001

a Data from study group 1, n = 56. We found no evidence of two-way interactions or non-linear effects using squared terms for these models. Age, gender, CF-related diabetes, airway infection with either *Pseudomonas aeruginosa* or *Staphylococcus aureus* and chronic azithromycin, oral or inhaled steroid use had no significant interactions with any inflammatory marker terms in any multivariate model. Log transformed values of biomarkers were used for modeling outcomes. Concurrent FEV_1_% and Weight-for-age *z*-score models used linear regression. The model for the number of APE occurring in the year prior to initial sputum collection used quasi-Poisson regression.

b Data from study group 2, n = 26. Additional adjustment for the stable FEV_1_% measurement, sequence of stable and APE time point collections, airway infection with either *Pseudomonas aeruginosa* or *Staphylococcus aureus*, use of azithromycin or steroids had no significant effect in these models.

c Estimates of the mean change in FEV_1_% per unit change in log scale biomarkers. Results from a linear regression model for the associations between difference in FEV_1_% between stable and APE time points and GM-CSF (log scale) measured at the APE onset time point. Each univariate representing measurements obtained during clinically stable and APE time points were added in turn to a model containing GM-CSF measured at the APE time point, the only statistically significant univariate. IL-5 (*p* = 0.006) and IL-10 (*p* = 0.015) measured at the APE time point and TCC (*p = *0.012) measured at the stable time point were found to be positively associated with FEV_1_% decline independently of GM-CSF. Backward selection of a multivariate model containing GM-CSF (APE), IL-5 (APE), IL-10 (APE), and TCC (Stable) produced the final model presented here.

d Estimates of the predicted total number of APE during 5 years of follow up per unit change in log scale biomarkers measured during clinical stability. Results show a quasi-Poisson regression model for the association with number of APE during 5 years of follow-up. HMGB-1 (log scale) was the only significant univariate (*p*<0.05), but CRP, IFN-α and IL-8 (all log scale) had trends toward significance (*p*<0.2). Backwards multivariate model selection retaining adjustment variables for follow-up time and low or high number of APE in the year prior to stable sputum collection (low  =  0 or 1 (reference group), high >1) as an indicator of baseline inflammation, retained only HMBG-1. A 1 unit change in log scale HMGB-1 is associated with a mean change in number of APE of 0.34.

### Analysis 2: Biomarker Behavior with an APE

We looked for biomarkers that changed in association with a clinical change from stable to APE state. For 19 of 22 biomarkers measured for Study Group 2 patients, the mean change between stable and APE states (Table S1) was positive indicating a non-random inflammatory state change (sign test, *p* = 0.006). C-reactive protein (CRP), IL-1β and IFN-α were statistically significantly higher (paired t-test, *p*<0.05) during an APE (Figure S1D-F). We investigated a potential effect of sample collection sequences (stable-then-APE and APE-then-stable) in sensitivity analyses. Mean IFN-α change was greater for the sequence stable-then-APE (*t*-test, *p* = 0.015). No sample-sequence-associated differences were found for CRP or IL-1β. (Discussion of these results can be found in Text S1.).

### Analysis 3: Relationship of Biomarkers with APE-associated Clinical Changes

For the 26 Study Group 2 patients, mean FEV_1_% decline between stable and APE time points was 7.3 (SD 7.8) percentage points; 5 had no decline. Univariate linear regressions found statistically significant inverse associations between FEV_1_% decline and GM-CSF, IL-23, CRP and sputum total cell count (TCC) each measured at APE time points and the difference in IL-17 measurements at stable and APE time points (Table S3). Neither biomarker measurements at stable time points nor any other biomarker differences between stable and APE time points were associated with FEV_1_% decline. After backward selection of multivariate models, GM-CSF and to a lesser extent IL-5, both measured at APE time points, were significantly and independently associated with APE-associated decline in FEV_1_% (Figure 1C–D, Table 5).

### Analysis 4: Predicting Future APE

Increased HMGB-1, adjusted for number of prior-year APE and follow-up time, was retained after backwards and forwards selection in a final multivariate quasi-Poisson regression model for the total number of APE occurring within 5 years of clinically-stable-time-point samples (Table 5). All other clinical measurements including *P aeruginosa* and *S aureus* infections, FEV_1_%, 5-year predicted survival [2] and other potential biomarkers measured at the stable-time point were not statistically significant predictors when forced into the final multivariate model (p = 0.23 for FEV_1_%; *p*>0.05 for all other covariates).

**Figure 2:**
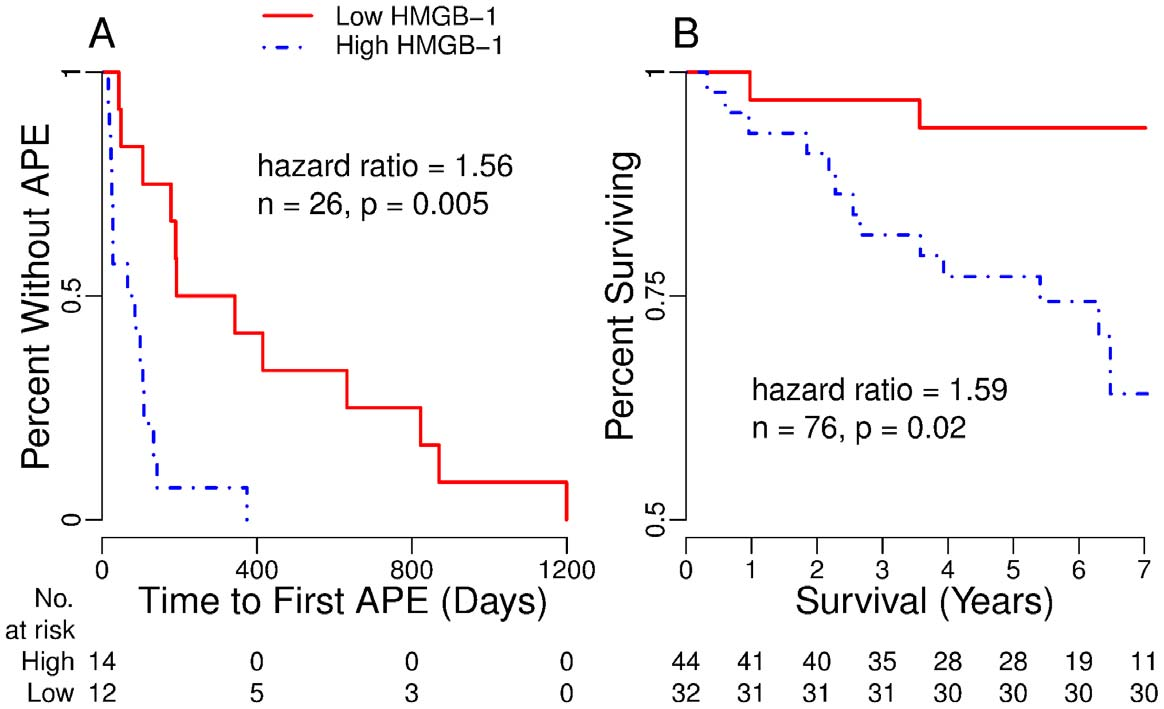
Kaplan-Meier Curves for the Time from Stable Sputum Collection to First Event. The curves illustrate the difference in time to A) first APE and B) death or censoring by listing for lung transplantation for patients with HMGB-1 measurements higher and lower than the value of 6.0 (log ng/ml). The value is the rounded median of the actual HMGB-1 data for both the 26 patients in A and the 76 patients in B. P-values shown are the results of log rank testing [52]. These graphs show the results of evaluation of HMGB-1 simplified to high or low values, which are consistent with the proportional hazards modeling [29] of the effects of HMGB-1 as a continuous variable. Models were tested for consistency with proportionality [31] (Table 6).

We have complete follow-up from clinically-stable time points to first APE, enabling proportional hazards modeling of time-to-first APE [29]–[31]. Univariate and multivariate models found HMGB-1 alone predicts time-to-first APE (Table 6, Figure 2A). Because of this result, we explored using median HMGB-1 level as a clinical test for predicting an APE within 6 months and found favorable preliminary results (see Text S1 and Figure S2).

**Table 6:** Proportional Hazards Models of Time-to-Event^a^.

Outcome	Biomarker	Study Group	Log Hazard Ratio	Standard Error	Hazard Ratio	95% Confidence Interval	*p*-value
First APE	HMGB-1	2	0.444	0.159	1.56	1.14 to 2.13	0.005
Lung Transplant or Death	HMGB-1	3	0.461	0.200	1.59	1.07 to 2.35	0.02

a The table shows results from proportional hazards models for the association between time-to-first APE following sputum collection and HMBG-1 (log scale) measurement from clinically-stable time points, Study Group 2, n = 26, and the association between time-to-lung transplant or death following initial sputum collection and HMGB-1 (log scale) measurements for all patients in the study with sufficient sample to measure HMGB-1, Study Group 3, n = 76. Both analyses shown met the assumption of proportionality for proportional hazards modeling [31]. Among the 76 patients, there were 15 events: 9 deaths and 6 listings for lung transplantation. All listed patients were subsequently transplanted. Adjustments for number of APE in the year prior to stable sputum collection were non-significant, and inclusion of variables for use of azithromycin or steroids had no effect on these models. Concurrent FEV_1_% and airway infection with either *Pseudomonas aeruginosa* or *Staphylococcus aureus* had non-significant associations with time-to-first APE. FEV_1_% is confounded as a predictor of time-to-transplant or death (See Discussion). *P aeruginosa* and *S aureus* infection are not primarily considered in selection of candidates for transplant and are not potential confounders; they had no effect on time-to-transplant or death. Approximately a 10% increase in HMGB-1 is associated with a 4% increase in the hazard rate for time-to-first APE and a 5% increase in hazard rate for time-to-lung transplant or death.

### Analysis 5: HMGB-1 as an Independent Predictor of Lung Transplantation or Death

Because of multiple other statistically significant associations, we assessed HMGB-1 ability to predict time-to-lung transplantation or death [29]–[31]. The composite outcome avoids 2 biases: 1) in a study of time-to-death, transplantation of end-stage patients creates an informative censoring bias and 2) in a study of time-to-lung transplantation, use of FEV_1_% to select transplantation candidates confounds the analysis. However, the composite outcome would still be confounded by modeling factors used to select transplantation candidates [2], [32], [33], thus potential covariates including FEV1% and prior-year APE were necessarily excluded from analysis. Other potential covariates including *P aeruginosa* and *S aureus* infection status do not alter lung transplantation candidacy and were included in the analysis. Follow-up time for the composite event was time-to-listing for transplantation, death, loss-to-follow-up or study end. Three patients lost-to-follow-up were treated as alive at the time of censoring. After multivariate proportional hazards modeling with backward and forward selection, we found that higher HMGB-1 values alone are strongly associated with shorter time-to-transplantation or death (Table 6, Figure 2B).

### Validation

Analyses 3–5 were repeated using data from four validation patient groups (Tables 1 and 2) to validate predictive abilities of HMGB-1 and GM-CSF. All validation results were similar to primary results. We evaluated results for mutual consistency using a weighted least-squares analysis and found that differences in coefficients and hazard ratios were insufficiently discrepant to cause concern (Table 7).

**Table 7:** Testing Validation Results for Mutual Consistency.

HMGB-1 Coefficients (SE)				
Outcome	Study Group Patients	Validation Group 1, n = 27	Validation Group 2, n = 17	Mutual Consistency Statistic^a^ (*p*-value)
Predicted APE during follow up	0.34 (0.08)^b^	0.25 (0.18)	0.11 (0.26)	0.78 (0.68)
First APE	0.44 (0.16)^b^	0.41 (0.18)	0.02 (0.30)	1.64 (0.45)
Lung Transplant or Death	0.46 (0.20)^c^	–	0.48 (0.52)^d^	9.4 × 10^−4^ (>0.99)
**GM-CSF Coefficients (SE)**				
**Outcome**	**Study Group Patients**	**Validation Group 3, n = 21**	**Validation Group 4, n = 13**	**Mutual Consistency Statistic** a **(** ***p*** **-value)**
APE-associated Decline in FEV_1_%	−8.48 (1.42)^b^	−4.69 (1.75)	−6.43 (5.70)	2.83 (0.25)

a Weighted least squares analysis.

b Study Group 2 patients, Analysis 3, n = 26.

c Study Group 3 patients, Analyses 4 and 5, n = 76.

d Validation Group 2 patients not included in Group 3, n = 9 with 1 death, 1 lung transplant.

## Discussion

Sputum HMGB-1 measurements predicted lung transplantation or death for CF patients during 7.1 years of follow-up (Table 6, Figure 2B). HMGB-1 measured during clinical stability surpassed FEV_1_% and all other biomarkers alone or in combination in ability to predict number of subsequent APE (Table 5) and time-to-first APE (Table 6, Figure 2A). Simultaneously, HMGB-1 measurements possess immediate clinical relevance by demonstrating strong independent associations with concurrent FEV_1_% and number of prior-year APE (Table 5) [2].

Our results confirm published findings from 32 CF patients that HMGB-1 inversely correlates with FEV_1_% and has an association with patients that suffer an APE. The earlier statistical findings were limited to concurrent clinical status [34]. Our study substantially extends these results finding evidence that HMGB-1 predicts future clinical events including survival (Tables 5 and 6).

HMGB-1 is required for normal cellular function but when released due to necrosis or secretion [9], [10] plays key roles in inflammatory arthritis [35], sepsis [11], and acute lung injury [36]. It mediates endotoxin-associated inflammation [10] through RAGE that is upregulated specifically in CF airways [7] and other receptors [37]. In rat models, HMGB-1-blocking antibodies reversed arthritis [35] and produced remarkably improved, durable survival despite delayed intervention following induction of septic shock by cecal ligation and puncture [10]. Increased HMGB-1 is associated with human acute lung injury [36] and sustained COPD-related inflammation [38]. In CF, HMGB-1 may increase pulmonary inflammation by attracting neutrophils [34], preventing their efferocytosis [39] and amplifying the effects of bacterial lipopolysaccharide and cytosine-phosphatidyl-guanosine-DNA constructs [40]. Soluble RAGE, an antagonist to HMGB-1-RAGE mediated inflammation, is absent in CF airways, potentiating HMGB-1 effects [7]. Our successful predictions (Tables 5 and 6, Figure 2) strengthen the argument that HMGB-1 may play a causal role in human inflammatory lung disease.

Low lung function shortens CF survival [2]. Sharp FEV1% declines define APE for most patients and may presage permanent lung function loss [5]. In this study, several biomarkers had significant univariate associations with acute APE-associated FEV_1_% decline, but GM-CSF and to a lesser extent IL-5 measured at APE time points together had the strongest association (Figure 1C–D, Table 5). These associations were independent of FEV_1_% itself and either cytokine measured during clinical stability. Our findings reflect previously known relationships in structure, expression and function of GM-CSF and IL-5, including the strong correlation between their measurements (Table S4) and the observation that one cytokine, in this case GM-CSF, is often more prominent than the other [41]. For GM-CSF at an APE time point, an approximate 1% increase was associated with a 10 point decline from the clinically-stable FEV1%. A patient’s GM-CSF measurement may quantify APE severity independently of prior clinical state and might provide laboratory-based objective support for hospitalization decisions.

GM-CSF maintains normal alveolar macrophage and innate immune responses to acute *P aeruginosa* pneumonia in mice [42]. In humans, elevated sputum GM-CSF is associated with increased COPD and asthma severity [43]. However, its role in chronically infected human airways is unclear. *P aeruginosa* or *S aureus* in CF airways [1] elicit increased epithelial GM-CSF secretion leading to prolonged survival and decreased apoptosis of airway neutrophils [44] and may prolong protease and reactive oxygen species releases [2].

Our definition of APE, based on experience and bedside utility, played a central role in defining events, sample collections and analysis of our major results with HMGB-1 and GM-CSF. It resembles prior definitions [2], [4], [15], but differs by excluding retrospective criteria such as a prior decision to give antibiotics [4] and by exchanging quantitative scoring [15] for bedside facility. Nevertheless our definition includes the key covariates of the existing quantitative model [15].

Our study focused on adult CF patients with moderately severe disease (Table 3), thus it provides no information easily extrapolated to children or mild or end-stage adult patients. However, we enrolled more than half of our large adult CF center and two-thirds of sputum producers. Our patients were nearly indistinguishable from the 70% of 2006 CFFPR adult patients nationally that produced sputum, a large, identifiable group that would benefit from new insights and therapies (Table 4) [1]. Volume of sputum collection could not be compared to the national population but had no effect in any of our analyses (not shown).

HMGB-1 was the key biomarker identified in multivariate models, proving to be superior to IL-8 and other biomarkers of previously established interest, indicating that HMGB-1 measurement alone is sufficient to report the information encoded in the other biomarkers evaluated (Table S1). However, we could not study all potentially significant biomarkers. To avoid compromising sample collection (see Text S1) [18], we excluded proteinases such as neutrophil elastase [45], [46], matrix metalloproteinase [47], [48] and proteinase-3 [49], [50] which may all cause lung tissue injury. Thus further studies are needed to explore the relative clinical predictive ability of these and still other biomarkers.

Our HMGB-1 and GM-CSF results successfully validated (Table 7) with additional but greatly dissimilar patients (Table 3). In every case, results were unaffected by commonly-prescribed potential modifiers of inflammation such as azithromycin and inhaled steroid therapies or by airway infections with *Pseudomonas aeruginosa* or *Staphylococcus aureus*. Nevertheless, this was still a single center study that was exploratory in nature, and collection of additional high-quality data from a larger cohort is needed to confirm our findings and explore directions of causality [51].

It is not feasible to include FEV_1_% or several other clinical covariates with HMGB-1 to predict time-to-lung transplantation or death due to biases from confounding and informative censoring. These biases arise because the same clinical covariates are used for selection of lung transplantation candidates. Prediction of either transplant or death alone is equally not feasible due to the same biases. Using a combined endpoint of lung transplant or death and excluding confounding variables avoided these biases but assumed that our clinical decisions [32] were accurate in identifying patients at highest risk of death [2]. These limitations are not specific to our study size, population or design. Rather, they apply to any population where lung transplantation is utilized. A positive byproduct results from avoiding these biases: this study focuses on causal pathways for airway inflammation leading to end-stage lung disease and avoids confusion from consideration of intermediate clinical findings like FEV_1_% that may not be causal [51].

This study identified airway-inflammatory biomarkers reporting on distinct pathophysiologic states that precede, accompany and follow an APE and provided clinically relevant information about permanent lung function losses on short and early mortality on long time scales. GM-CSF and HMGB-1 provide potentially useful new measurements for monitoring short and longer term treatment effects for CF. Both biomarkers provide focal points for additional clinical, epidemiologic and mechanistic investigations of CF airway inflammation. In particular, HMGB-1 provides pathophysiologic information predictive of APE and survival that might guide preemptive treatment and provide a novel target for therapy.

## Supporting Information

Figure S1 **Biomarker Relationships with Concurrent Clinical Outcomes and Biomarker Changes between Stable and APE States.** Relationships between A) IL-17A and concurrent FEV_1_%, B) IL-17A and weight-for-age *z*-score and C) IFN-α with prior year APE. Three biomarkers, D) CRP, E) IL-1β and F) IFN-α are increased in sputum collected at the time of diagnosis of an APE compared to their measurements during clinical stability using paired *t*-tests. Red symbols illustrate values for patients that underwent the sputum collection sequence stable-then-APE while blue symbols denote patients that underwent the sequence APE-then-stable. The number of days between stable and APE sputum collections had no effect on differences on measurements (multiple linear regression *p*>0.5). However, changes in these three biomarker measurements are unsuitable for clinical use to identify an APE due to substantial overlap of APE and stable time point values.
(EPS)

Figure S2 **Receiver operator characteristic curves for potential markers of an APE within 6 months.** Receiver operator characteristic curves were constructed for 9 biological or clinical markers of CF using APE within 6 months as the clinical discriminator. Log-scale biomarker values were derived from measurements of sputum samples from 26 patients during clinical stability (Study Group 2, Tables 1–2). Each plot has a color bar presenting the range of cut-off values of each biomarker used to create the points on each ROC curve. The area under the curve (AUC or accuracy) and 95% confidence interval for each AUC reported for each curve was the result of boot strapping of the AUC using 1,000 repetitions. HMGB-1 has the highest accuracy of any marker studied.
(EPS)

Table S1 **Sputum Biomarker Measurements.**
(DOC)

Table S2 **Univariate Associations of Biomarkers with Clinically Relevant Concurrent Conditions.**
(DOC)

Table S3 **Univariate Associations of Biomarkers for APE-associated FEV_1_% drop.**
(DOC)

Table S4 **Correlations (p-values) between biomarkers, Study Group 1.**
(DOC)

Text S1 **Detailed Laboratory and Statistical Methods, Additional Results and Discussion.**
(DOC)
