# Supplementary material for: Sputum Biomarkers and the Prediction of Clinical Outcomes in Patients with Cystic Fibrosis
Source: PLoS One. 2012 Aug 10;7(8):e42748. doi: 10.1371/journal.pone.0042748 (PMC3416785; doi:10.1371/journal.pone.0042748)
Supplement: Table S1 — Sputum Biomarker Measurements. (DOC) [file pone.0042748.s003.doc]

Table S1. Sputum Biomarker Measurements

| **Biomarker^b^** | **Study Group** **1, n = 56** | **Study Group 2^a^, n = 26** | | | **Study Group 3,**  **n = 76** |
| --- | --- | --- | --- | --- | --- |
|  | **Mean (SD)^c^** | **Mean (SD)^c^** | | **Paired *t*-test *p*-value** | **Mean (SD)^c^** |
|  |  | **Stable** | **APE** |  |  |
| CRP | – | 12.5 (1.61) | 13.9 (1.70) | <0.001 | – |
| G-CSF | 5.40 (1.22) | 4.46 (1.77) | 4.8 (1.26) | 0.21 | – |
| GM-CSF | 4.66 (0.96) | 2.94 (0.81) | 3.05 (0.71) | 0.59 | – |
| HMGB-1 (log ng/ml) | 5.54 (1.73) | 6.16 (1.39) | 6.49 (1.85) | 0.45 | 5.84 (1.75) |
| IL-1β | 7.61 (0.90) | 7.47 (1.24) | 8.01 (1.39) | 0.016 | – |
| IL-2 | 0.007 (0.68) | 1.97 (0.80) | 2.13 (0.56) | 0.37 | – |
| IL-5 | -0.18 (0.69) | -0.65 (0.40) | -0.46 (0.52) | 0.13 | – |
| IL-6 | 7.21 (1.05) | 3.30 (1.33) | 3.36 (1.11) | 0.80 | – |
| IL-8 | 10.0 (0.94) | 12.8 (0.48) | 12.8 (0.51) | 0.70 | – |
| IL-10 | 2.29 (0.80) | 1.17 (0.99) | 1.37 (1.05) | 0.37 | – |
| IL-12p40 | 1.09 (1.02) | – | – | – | – |
| IL-17A | 0.62 (1.56) | 4.19 (0.76) | 4.44 (0.81) | 0.29 | – |
| IL-23 | – | 4.36 (1.02) | 4.79 (1.20) | 0.16 | – |
| IFN-α | 2.31 (0.54) | 0.41 (0.93) | 1.05 (1.20) | 0.03 | – |
| IFN-γ | -0.83 (1.25) | -2.04 (0.96) | -1.53 (1.41) | 0.11 | – |
| MBL | – | 7.61 (1.56) | 8.09 (1.55) | 0.13 | – |
| MCP-1 | – | 5.98 (1.16) | 5.55 (0.86) | 0.071 | – |
| MIP-1α | 4.66 (0.30) | 2.36 (1.59) | 1.91 (1.34) | 0.17 | – |
| MPO | 15.5 (0.91) | 15.3 (0.087) | 15.2 (0.15) | 0.25 | – |
| sRAGE | – | 2.15 (0.85) | 2.45 (0.76) | 0.15 | – |
| TGF-β_1_ | 6.65 (0.60) | 5.03 (0.93) | 5.65 (1.17) | 0.052 | – |
| TNF-α | 4.49 (0.49) | 2.77 (1.01) | 2.85 (0.93) | 0.69 | – |
| TCC (log cells per ml) | 17.2 (0.75) | 17.0 (0.88) | 17.3 (0.96) | 0.21 | – |

^a^ All values shown are pg/ml except as noted and are log transformed. Patients in Study Group 2 provided two samples each: one during clinical stability and one at the onset of hospitalization for an APE; *p*-values reported are for paired *t-*tests.

^b^ Abbreviations Used: CRP: C-reactive protein, G-CSF: granulocyte-colony stimulating factor, GM-CSF: granulocyte macrophage colony stimulating factor, HMGB-1: high mobility group box-1 protein, IL: interleukin, IFN-α: interferon-alpha, IFN-γ: interferon-gamma, MBL: mannose binding lectin, MCP-1: macrophage chemotactic protein-1, MIP-1α: macrophage inhibitory protein-1-alpha, MPO: myeloperoxidase, sRAGE: soluble receptor for advanced glycation end-products, TGF-β_1_: tissue growth factor-beta-1, TNF-α: tumor necrosis factor-alpha, TCC: total cell count.

^c^ Standard Deviation (SD). For some biomarkers, some samples had undetectable results (UD). We assigned half the lowest detected value to UD measurements for log transformation. In preliminary measurements, most values of IL-3, IL-4 and IL-11 were undetectable (see Methods). Unmeasured values are denoted by dashes. All values are log-transformed from pg/ml except as noted for HMGB-1 and TCC.
