# Supplementary material for: Sputum Biomarkers and the Prediction of Clinical Outcomes in Patients with Cystic Fibrosis
Source: PLoS One. 2012 Aug 10;7(8):e42748. doi: 10.1371/journal.pone.0042748 (PMC3416785; doi:10.1371/journal.pone.0042748)
Supplement: Table S2 — Univariate Associations of Biomarkers with Clinically Relevant Concurrent Conditions. (DOC) [file pone.0042748.s004.doc]

Table S2. Univariate Associations of Biomarkers with Clinically Relevant Concurrent Conditions

| **Biomarker** | **Regression Coefficients (*p*-value)^a^** | | | | |
| --- | --- | --- | --- | --- | --- |
|  | **FEV_1_%** | **Weight-for-age *z*-score** | **APE** | **Chronic Airway Infection** | |
|  |  |  |  | ***P aeruginosa*** | ***S aureus*** |
| **G-CSF** | **10.0 (<0.001)** | 0.16 (0.097) | -0.19 (0.021) | -0.088 (0.061) | 0.14 (0.006) |
| **GM-CSF** | 7.5 (0.038) | 0.014 (0.91) | -0.07 (0.51) | -0.059 (0.34) | 0.18 (0.004) |
| **HMGB-1** | **-8.5 (<0.001)** | -0.21 (0.0041) | **0.2 (0.001)** | 0.015 (0.67) | -0.016 (0.68) |
| **IL-1β** | -4.2 (0.27) | -0.12 (0.36) | 0.21 (0.066) | 0.07 (0.27) | 0.084 (0.23) |
| **IL2** | 12 (0.019) | 0.12 (0.47) | -0.051 (0.74) | 0.022 (0.8) | 0.13 (0.17) |
| **IL5** | 0.55 (0.91) | 0.1 (0.54) | 0.12 (0.42) | 0.07 (0.4) | -0.097 (0.28) |
| **IL6** | -5.3 (0.11) | -0.15 (0.18) | -0.0079 (0.94) | 0.0037 (0.95) | -0.022 (0.72) |
| **IL8** | -9.7 (0.0067) | -0.029 (0.81) | 0.28 (0.008) | 0.17 (0.005) | -0.12 (0.061) |
| **IL10** | 0.06 (0.99) | -0.038 (0.79) | 0.13 (0.31) | 0.082 (0.26) | 0.022 (0.78) |
| **IL12p40** | 3.5 (0.3) | -0.055 (0.62) | 0.033 (0.74) | -0.0071 (0.9) | 0.028 (0.65) |
| **IL13** | 0.11 (0.38) | -0.0029 (0.48) | -1.8e-05 (0.99) | -0.0011 (0.6) | 0.0015 (0.52) |
| **IL17** | **10.0 (<0.001)** | **0.23 (<0.001)** | **-0.24 (<0.001)** | -0.044 (0.23) | 0.033 (0.41) |
| **IFN-α** | 6.1 (0.34) | -0.13 (0.53) | -0.29 (0.13) | 0.03 (0.78) | -0.036 (0.76) |
| **IFN-γ** | 5.3 (0.054) | -0.11 (0.24) | -0.0071 (0.93) | -0.066 (0.15) | 0.016 (0.76) |
| **MIP-1α** | 28 (0.012) | 0.49 (0.2) | -0.43 (0.2) | -0.019 (0.92) | -0.18 (0.39) |
| **MPO** | **-12 (<0.001)** | -0.16 (0.21) | 0.27 (0.014) | 0.074 (0.24) | -0.043 (0.54) |
| **TGF-β** | -1.5 (0.79) | -0.28 (0.15) | 0.15 (0.38) | 0.096 (0.32) | 0.083 (0.43) |
| **TNF-α** | 4.7 (0.52) | -0.17 (0.46) | -0.1 (0.63) | 0.15 (0.21) | -0.18 (0.17) |
| **TCC** | -8.7 (0.048) | -0.28 (0.056) | 0.37 (0.003) | 0.079 (0.29) | 0.15 (0.06) |

^a^ Study Group 1, n = 56. For FEV_1_% and BMI: linear regression coefficients using log-transformed biomarker measurements. For APE: regression coefficients from quasi-Poisson regressions using log-transformed biomarker measurements. For *S aureus* and *P aeruginosa*: log odds ratios from logistic regressions using log-transformed biomarker measurements. Grey text identifies non-significant results. Normal text identifies results with significant uncorrected *p*-values (*p* < 0.05). **Bold text** identifies results that remain significant after stringent Bonferroni correction (*p* < 0.0026).
