# Supplementary material for: Sputum Biomarkers and the Prediction of Clinical Outcomes in Patients with Cystic Fibrosis
Source: PLoS One. 2012 Aug 10;7(8):e42748. doi: 10.1371/journal.pone.0042748 (PMC3416785; doi:10.1371/journal.pone.0042748)
Supplement: Table S3 — Univariate Associations of Biomarkers for APE-associated FEV1% drop. (DOC) [file pone.0042748.s005.doc]

**Table S3. Univariate Associations of Biomarkers for APE-associated FEV_1_**% drop

|  | **Stable Measurement** | | **APE Measurement** | | **Stable-APE Measurement Differences** | |
| --- | --- | --- | --- | --- | --- | --- |
| **Biomarker** | **Coefficient (SD)** | ***p*-value** | **Coefficient (SD)** | ***p*-value** | **Coefficient (SD)** | ***p*-value** |
| GM-CSF | -0.35 (1.41) | 0.86 | **-8.48 (1.19)** | **3.64 × 10^-6^** | -3.86 (1.16) | 0.008^a^ |
| IFN-γ | 0.066 (1.29) | 0.97 | -0.57 (1.06) | 0.62 | -0.48 (1.01) | 0.64 |
| IL-1β | -0.51 (1.14) | 0.70 | -1.54 (1.05) | 0.18 | -1.86 (1.19) | 0.20 |
| IL-2 | -1.48 (1.41) | 0.46 | -4.37 (1.65) | 0.12 | -0.51 (1.34) | 0.78 |
| IL-5 | 4.23 (1.99) | 0.30 | -1.87 (1.76) | 0.55 | -3.03 (1.59) | 0.24 |
| IL-6 | -0.14 (1.1) | 0.91 | -0.062 (1.20) | 0.97 | 0.11 (1.13) | 0.93 |
| IL-8 | -0.34 (1.82) | 0.92 | 1.71 (1.76) | 0.59 | 1.32 (1.59) | 0.60 |
| IL-10 | 0.71 (1.27) | 0.66 | 1.24 (1.23) | 0.42 | 0.53 (1.19) | 0.71 |
| TNF-α | 0.75 (1.25) | 0.64 | 0.021 (1.31) | 0.99 | -0.80 (1.28) | 0.63 |
| MCP-1 | 0.24 (1.17) | 0.86 | -1.55 (1.35) | 0.41 | -1.09 (1.17) | 0.43 |
| MIP-1α | 0.18 (1.00) | 0.86 | 1.06 (1.09) | 0.38 | 0.56 (1.00) | 0.58 |
| G-CSF | -0.19 (0.95) | 0.83 | -0.33 (1.13) | 0.80 | 0.044 (1.08) | 0.97 |
| IFN-α | -1.05 (1.3) | 0.54 | -2.45 (1.11) | 0.06 | -1.32 (1.05) | 0.24 |
| IL-17 | 2.86 (1.43) | 0.17 | -3.55 (1.36) | 0.07 | -3.00 (1.12) | 0.02 |
| IL-23 | -0.44 (1.25) | 0.78 | -3.27 (1.07) | 0.009 | -1.93 (1.00) | 0.06 |
| CRP | 0.094 (1.00) | 0.92 | -1.94 (0.92) | 0.032 | -1.91 (0.91) | 0.03^a^ |
| MPO | -31.1 (4.16) | 0.08 | -14.0 (3.24) | 0.19 | -2.32 (3.03) | 0.80 |
| RAGE | 0.42 (1.37) | 0.82 | -0.78 (1.44) | 0.71 | -0.72 (1.25) | 0.65 |
| TGF-β_1_ | 0.57 (1.31) | 0.74 | -2.0 (1.14) | 0.14 | -1.39 (1.00) | 0.18 |
| HMGB-1 | 0.11 (1.07) | 0.93 | -0.63 (0.92) | 0.47 | -0.13 (0.76) | 0.82 |
| MBL | -0.20 (1.01) | 0.86 | 0.11 (1.02) | 0.92 | 0.29 (1.01) | 0.78 |
| TCC | 0.50 (1.34) | 0.78 | -4.13 (1.19) | 0.008 | -3.82 (1.14) | 0.007^a^ |

Grey text identifies non-significant results. Normal text identifies results with significant uncorrected *p*-values (*p* < 0.05). **Bold text** identifies results that remain significant after stringent Bonferroni correction (*p* < 0.001). After Bonferroni correction*,* only the univariate result for GM-CSF measured in the APE state was significantly associated with APE-associated FEV_1_% drop.

^a^ The apparent significance of association of the difference in measurements between Stable and APE states for GM-CSF, CRP and TCC are all explained by the associations of the APE state measurements for these biomarkers with the APE-associated FEV_1_% drop.
