# Supplementary material for: Sputum Biomarkers and the Prediction of Clinical Outcomes in Patients with Cystic Fibrosis
Source: PLoS One. 2012 Aug 10;7(8):e42748. doi: 10.1371/journal.pone.0042748 (PMC3416785; doi:10.1371/journal.pone.0042748)
Supplement: Table S4 — Correlations (p-values) between biomarkers, Study Group 1. (DOC) [file pone.0042748.s006.doc]

**Table S4.** **Correlations (p-values) between biomarkers, Study Group 1^a^**

| **n = 56** | **GM-CSF** | **IL-1b** | **IL-2** | **IL-5** | **IL-6** | **IL-8** | **IL-10** | **IL-12p40** | **IL-13** | **IL-17** | **IFN-α** | **IFN-γ** | **MIP1-α** | **MPO** | **TGF-β** | **TNF-α** | **TCC** | **HMGB-1**^b^ |
| --- | --- | --- | --- | --- | --- | --- | --- | --- | --- | --- | --- | --- | --- | --- | --- | --- | --- | --- |
| G-CSF | **0.63 (1.8****×10-7)** |  | 0.35 (0.0078) |  |  | -0.37 (0.0055) |  |  |  | **0.72 (4.2×10^-10^)** |  | 0.38 (0.0044) | 0.38 (0.0042) | **-0.49 (0.00011)** |  |  |  | -0.3 (0.038) |
| GM-CSF |  |  | **0.81 (2.3×10^-14^)** | **0.5 (9×10^-5^)** |  |  |  | **0.66 (3.2×10^-8^)** | 0.4 (0.002) | 0.27 (0.046) |  | 0.41 (0.0016) |  |  |  |  |  |  |
| IL-1b |  |  | 0.36 (0.007) | 0.33 (0.014) |  | **0.56 (6.4×10^-6^)** | 0.41 (0.0019) |  |  | -0.32 (0.016) | -0.27 (0.043) |  | -0.43 (0.0011) | **0.49 (0.00013)** | **0.58 (2.9×10^-6^)** |  | **0.55 (1.2×10^-5^)** | 0.39 (0.006) |
| IL-2 |  |  |  | **0.6 (1.3×10^-6^)** |  |  |  | **0.7 (2.1×10^-9^)** | 0.43 (0.00094) |  |  | 0.38 (0.0038) |  |  |  |  |  | -0.38 (0.0072) |
| IL-5 |  |  |  |  |  |  |  | **0.48 (0.00019)** | 0.45 (0.00045) |  |  |  |  |  | 0.27 (0.041) |  |  |  |
| IL-6 |  |  |  |  |  | 0.4 (0.0025) |  |  |  |  |  |  |  | **0.47 (0.00027)** |  | 0.29 (0.03) |  |  |
| IL-8 |  |  |  |  |  |  | 0.31 (0.022) |  |  | -0.43 (0.00088) |  |  |  | **0.73 (1.6×10^-10^)** | **0.47 (0.00029)** |  | **0.47 (0.00029)** | 0.45 (0.0013) |
| IL-10 |  |  |  |  |  |  |  |  |  |  |  |  |  |  | 0.39 (0.0033) | **0.6 (9.1×10^-7^)** |  | 0.4 (0.0046) |
| IL-12p40 |  |  |  |  |  |  |  |  | **0.49 (0.00013)** |  |  | 0.35 (0.0078) |  |  |  |  |  |  |
| IL-13 |  |  |  |  |  |  |  |  |  |  |  | 0.27 (0.041) |  |  |  |  |  |  |
| IL-17 |  |  |  |  |  |  |  |  |  |  |  |  | **0.5 (0.0001)** | **-0.6 (1.3×10^-6^)** |  |  | -0.32 (0.017) | -0.46 (0.00097) |
| IFN-α |  |  |  |  |  |  |  |  |  |  |  |  | 0.4 (0.002) |  |  | **0.56 (7.3×10^-6^)** |  |  |
| IFN-γ |  |  |  |  |  |  |  |  |  |  |  |  |  |  |  |  |  |  |
| MIP1-α |  |  |  |  |  |  |  |  |  |  |  |  |  | -0.3 (0.022) |  | 0.44 (0.0006) | -0.31 (0.02) | -0.34 (0.018) |
| MPO |  |  |  |  |  |  |  |  |  |  |  |  |  |  | 0.4 (0.002) |  | **0.58 (2.4×10^-6^)** | **0.53 (0.0001)** |
| TGF-β_1_ |  |  |  |  |  |  |  |  |  |  |  |  |  |  |  |  | 0.46 (0.00031) | 0.35 (0.015) |
| TNF-α |  |  |  |  |  |  |  |  |  |  |  |  |  |  |  |  |  |  |
| TCC |  |  |  |  |  |  |  |  |  |  |  |  |  |  |  |  |  | 0.31 (0.029) |

^a^ Only significant correlations (Pearson's Product Moment Test) are shown (*p* < 0.05). Correlations significant after stringent Bonferroni correction (*p* < 0.0003) are shown with **bold text**.

^b^ These correlations used data from 49 patients due to missing data for HMGB-1 for 7 individuals.
