# Supplementary material for: Sputum Biomarkers and the Prediction of Clinical Outcomes in Patients with Cystic Fibrosis
Source: PLoS One. 2012 Aug 10;7(8):e42748. doi: 10.1371/journal.pone.0042748 (PMC3416785; doi:10.1371/journal.pone.0042748)
Supplement: Text S1 — Detailed Laboratory and Statistical Methods, Additional Results and Discussion. (DOC) [file pone.0042748.s007.doc]

**On-line Supplement**

*Details on Patient Selection for main study or validation groups*

Patients were enrolled in this study if they gave signed consent and expectorated sputum. The main investigative group of patients was enrolled from March 2004 through April 2007. Out of these 85 patients, after feasibility estimates based primarily on cost of biomarker assays, 80 patients were randomly chosen using Patient ID numbers. Of these 80 patients, 56 had multiplex Searchlight assays and are described in Table 3 as Study Group 1; an additional 24 patients were selected for multiplex assay and to be part of Study Group 1, but simultaneous separate shipment of their specimens thawed prior to testing resulting in no usable measurements. Study Group 1 Searchlight, HMGB-1 and GM-CSF sputum assay results are given in Table S1, first column. Out of the 85 main investigative group of patients, 26 produced two sputums: one from a stable state and one from an APE state that were adjacent in time. These 26 patients form Study Group 2; the 52 sputum samples from Study Group 2 underwent a second Searchlight multiplex assay as a single batch that is reported in Table S1, 2^nd^ and 3^rd^ columns. Finally, out of the 85 patients, a total of 76 patients, Study Group 3, underwent HMGB-1 assays as reported in Table S1, last column. Study Groups 1, 2 and 3 included all of the patients recruited March 2004 through April 2007.

Patients that gave consent during March 2004 through April 2007 that provided sputum samples that were collected when clinically stable or with mild exacerbation (a clinical presentation typically with symptoms but without objective findings that did not require hospitalization) that did not provide an accompanying sputum from an APE and had HMGB-1 measured during the same time period as Study Group 2 samples form Validation Group 1. These 27 patients are included in All Patients, and are non-overlapping with Study Group 2 (Tables 1-3).

Patients that gave consent and a sputum during a stable or mild exacerbation states March, 2004 through 2011 for ongoing sputum collections and had HMGB-1 measurements in 2011 form Validation Group 2 and are non-overlapping with Study Group 2 (Tables 1-3).

Patients that gave consent during March 2004 through April 2007 and gave a sputum during an APE and were not part of Study Group 2 but had their sputum GM-CSF measured at the same time form Validation Group 3. Patients that gave consent for ongoing sputum collections through 2011, gave sputum during an APE and had GM-CSF measured during 2011 form Validation Group 4. While there is no overlap in patients between Study Group 2 and Validation Group 4, there were 9 patients in Study Group 3 that were also in Validation Group 4. The validation of Analysis 5 using Validation Group 4 excludes those 9 overlapping patients (See Tables 1-3 and 7 for details).

*Sputum Processing*

We collected sputum samples in clinic, immediately placed samples on ice and added anti-protease cocktail (Sigma, St. Louis, MO) to prevent neutrophil proteinase-mediated biomarker degradation [1]. We avoided dithiothreitol because of previously reported negative effects on cytokine measurements [2]. After assessing salivary contamination, we performed total cell counts (TCC) and differentials using modified Wright staining. Sputum was diluted 1:4 with Hanks Buffered Saline Solution (Sigma), vortexed and centrifuged to separate aqueous supernatants from pellets and lipid top layers. Supernatants were frozen in small aliquots at -70 °C until analyzed.

We measured potential biomarkers previously identified as important in CF [1,3–11] using SearchLight multiplex assay services (Aushon Biosystems, Billerica, MA). We measured granulocyte colony stimulating factor (G-CSF), granulocyte macrophage colony stimulating factor (GM-CSF) , interferon-(IFN)-α, IFN-γ, interleukin-(IL)-1β, IL-2, IL-5, IL-6, IL-8, IL-17A, myeloperoxidase (MPO), macrophage inflammatory protein-1-α (MIP-1-α ), transforming growth factor-β_1_ (TGF-β_1_), tumor necrosis factor-α (TNF-α), C-reactive protein (CRP), IL-23, monocyte chemotactic protein-1 (MCP-1), and soluble receptor for advanced glycation end-products (sRAGE). We found IL-3, IL-4, IL-11 undetectable. We measured HMGB-1 and MBL by ELISA using commercially-available antibodies (R&D Systems, Inc, Minneapolis, MN) and previously published protocols [12,13] after confirming assay reproducibility on consecutive days.

*Comparison of Study Patients with the 2006 CF Foundation Patient Registry*

To investigate the generalizability of our results, we compared the specific characteristics of our study patients with similar patients in the CFFPR. Three covariates with the potential to modify our results, azithromycin, inhaled and oral steroid use, were recorded in the CFFPR beginning in 2006, the last complete year in our enrollment period. We thus compared our patients with patients from 2006.

There were 20,047 patients in the CFFPR in 2006. Of these, 10,004 were adults: 7,593 were sputum producers, 843 were non-sputum producers and 2,411 had no information regarding sputum production. χ-square analysis showed that our sputum producing patients constituted either a significantly larger or smaller proportion of clinic patients able to produce sputum depending on the number of sputum producers in the unspecified group in the CFFPR suggesting no significant difference. Similarly, we performed χ-square analysis for gender distribution, *Pseudomonas aeruginosa* and *Staphylococcus aureus* infections, use of azithromycin, inhaled and oral steroids. Our patients were not statistically different from patients in the CFFPR except for use of oral steroids. We found a lower use of oral steroids among our patients (Table 4), but usage had no effect on our results (Tables 5 and 6). For continuous variables, we performed comparisons with the Student's *t*-test for normally distributed or the Kolmogorov-Smirnov test for non-normally distributed continuous variables [14]. We found a significant but small difference in weight-for-age *z*-score (Table 4). However, this had no impact on our main results involving HMGB-1 and GM-CSF (Tables 5 and 6).

*Statistical Analysis*

We examined (1) associations between inflammatory markers and concurrent clinical measures of disease, (2) the effect of an APE on inflammatory marker levels, (3) the ability of inflammatory markers to predict clinical outcomes relevant to patients and clinicians. Following univariate analyses, we performed multivariate analyses with backward selection procedures in order to discover the most parsimonious models that still provided significant and informative fits for each of the outcomes selected. We followed backward selection with forward selection to ensure that important covariates were correctly excluded with backward selection. Forward selection, however, did not find that any of the covariates previously eliminated in backward selection made a significant contribution to final models. All statistical analyses were performed using R [15].

In Analysis 1, we used linear regression to assess univariate and multivariate associations for inflammatory biomarkers with FEV_1_% and weight-for-age *z*-score, quasi-Poisson regression to assess associations with APE in the year prior to sputum collection, and logistic regression to assess associations with the presence of bacterial infections. For analysis, biomarker measurements were transformed to the log scale following graphical investigations of relationships. All reported *p*-values are uncorrected for multiple comparisons, however in interpretations we assessed *p*-values for significance after stringent Bonferroni correction (Table S2).

We looked for between biomarker correlations (Pearson's Product Moment test) to help interpret results of multivariate modeling with clinical measurements (Table S4). Stringent Bonferroni correction was applied to identify correlations that might have affected interpretation of the multivariate models reported in Table 5, however, none of the associations proved necessary to consider in the interpretation of models involving HMGB-1. The correlation between GM-CSF and IL-5 measured during an APE was significant even after stringent Bonferroni correction.

In the multivariate models for each clinical finding (Table 5), we first omitted all univariates with an uncorrected *p* > 0.1. We then considered correlations between the remaining biomarkers in each multivariate model to select a model. Biomarkers not included in this model were added back in one by one to check whether their inclusion affected the coefficients associated with the selected biomarkers. Following candidate model derivations, we assessed the possibility of interactions and non-linear associations by testing all two-way interactions and squared terms. Where multiple covariates were previously noted to be moderately or strongly correlated, we examined coefficients for concordance with univariate effects. If two correlated variables (Table S4) were both in a multivariate model, we tested for spurious effects by eliminating both and examining log likelihoods. This proved necessary for the multivariate model for drop in FEV_1_% as a function of GM-CSF and IL-5 (both measured during an APE state, Table 5). After examining log likelihoods, both covariates were retained.

Analysis 2 compared biomarker levels obtained during clinical stability and at the onset of an APE (paired *t*-test) to assess the ability of each biomarker to indicate an APE. Using univariate linear regression models, we investigated the association between the FEV_1_% difference between stable and APE time point with biomarker measurements (log-scale) made at the same time points. All biomarkers with potentially significant relationships were entered in a multivariate linear regression model, and we used backwards model selection, excluding variables with *p*-value exceeding 0.05. We performed forward selection of remaining biomarkers to derive the final model, retaining variables with *p*-value less than 0.05. We investigated additional adjustment for FEV_1_% measured at the stable time point, and indicators of use of anti-inflammatory treatments.

In Analysis 3, quasi-Poisson regression was used to model the association between number of APE during follow-up and log biomarker measurements. All models were adjusted for length of follow-up, an indicator of the number of APE in the year prior to the stable sputum collection (low = 0 to 1, or high > 1) as a clinical indicator of baseline inflammation and the concurrent FEV_1_% measurement. Biomarkers were first assessed individually, and those with a potentially predictive relationship (*p*-value < 0.05) were entered in a further multivariate model. Backwards and forwards variable selection procedures were used to arrive upon the final model.

In Analysis 4, univariate proportional hazards models [16,17] were used to estimate hazard ratios for the association between biomarkers and time-to-first APE following the stable sputum collection. We entered potentially predictive variables into multivariate proportional hazard models and performed model selection. Finally, in Analysis 5, we used proportional hazards modeling [16,17] to examine the association between HMGB-1 levels and time-to-lung transplantation or death following the initial sputum collection for enrolled patients during follow-up.

*Additional Biomarker Measurements and Clinical Status*

Multiplex assays showed that 18 of 21 selected sputum biomarkers and HMGB-1 were measurable for Study Group 1 (Table S1). IL-3, IL-4 and IL-11 were undetectable in 53, 49 and 52 of the 56 samples from Study Group 1, respectively, excluding these biomarkers from further evaluation.

TCC averaged 4.63×10^7^ per ml. Neutrophils constituted more than 50% of cells for all samples with all but two having more than 90% neutrophils in their sputum.

We investigated collinearity between biomarkers by calculating all two way correlations (Table

S4). Out of 171 potential correlations, 64 were statistically significant (*p* < 0.05), and 25 remained so after stringent Bonferroni correction (*p* < 0.0003).

We looked for univariate associations with concurrent FEV_1_%, BMI, APE and culture evidence of *S aureus* or *P aeruginosa* (Table S2) and with APE-associated FEV_1_% decline (Table S3). Decreasing G-CSF, GM-CSF, IL-2, IL-17 and MIP-1α and increasing HMGB-1, IL-8, MPO and TCC were significantly associated with lowered FEV_1_%. Decreasing IL-17 and increasing HMGB-1 were significantly associated with lowered weight-for-age *z*-score (Table S2). Higher HMGB-1, IL-8, MPO and TCC and lower G-CSF and IL-17 were significantly associated with greater numbers of APE. IL-8 alone had a significant association with concurrent *P aeruginosa* infection while G-CSF and GM-CSF had significant associations with *S aureus* infection; however, no association with a bacterial infection survived stringent Bonferroni correction. Only GM-CSF measured during an APE was associated with APE-associated decline in FEV_1_% after stringent Bonferroni correction (Table S3).

Multivariate analysis and model selection using biomarkers which had a significant or borderline significant association in univariate models produced three final models for concurrent FEV_1_%, weight-for-age *z*-score and prior-year APE (Table 5). Univariate relationships between HMGB-1, IL-17, IFN-α and the three clinical characteristics are shown (Figure 1A-B and Figure S1A-C).

Additional analysis was performed looking for an association between biomarker levels and future FEV_1_%. No significant associations were discovered.

*HMGB-1 as a clinical predictor of APE within 6 months*

Because HMGB-1 was strongly associated with time-to-first APE in Study Group 2 patients, we evaluated the use of sputum HMGB-1 measured at the stable time point as a clinical test for prediction of an APE within 6 months. Using the approximate median value of 6.0 (log ng/ml) as a cut-off point, we found that HMGB-1 had a high positive predictive value (0.93), high specificity (0.89), moderately high sensitivity (0.76) and low negative predictive value (0.67) for an APE within 6 months of sputum collection.

We evaluated the receiver-operator characteristic (ROC) of HMGB-1, with APE within 6 months as the clinical discriminator [18]. The area under the ROC curve (AUC or accuracy) was estimated by bootstrapping with 1,000 iterations (Figure S2) [19]. HMGB-1 had a high AUC (0.88, 95% confidence interval = 0.73-0.99). We investigated IFN-α and IL-17A in the same way, because they were associated with time-to-first APE in proportional hazards modeling, though not statistically significantly so, and CRP and IL-1β, because they were elevated with an APE (Figure S1D-E). Clinicians currently use FEV_1_% and number of prior APE as estimators of disease severity, thus we also estimated ROC curves and AUC with 95% confidence intervals for each of these. Finally, we examined IL-8 because of prior high levels of interest in this factor [20] and TGF-β_1_ because of potential pathophysiologic interest in CF [7] (Figure S2).

HMGB-1 exhibited the best accuracy as a clinical test (AUC = 0.88), however, the number of Study Group 2 patients led to wide confidence intervals. The 95% confidence intervals for AUC associated with HMGB-1 measurements showed clear overlap with CRP, IL-1β, TGF-β_1_ and prior APE and marginal overlap with IL-17A, IFN-α. There was no overlap with IL-8 or baseline FEV_1_%.

These results suggest that HMGB-1 has potential for use as a clinical test. However, we had few patients for this type of analysis. Further prospective testing using the definition of APE that we presented herein with incidence within 6 months as the test outcome with larger number of patients should be performed. The results may be helpful to confirm the use of HMGB-1 measurement in CF.

*Biomarker Behavior with an APE*

Analysis 2 found three biomarkers that significantly increased in mean with an APE (Figure S1A-C, Table S1): CRP, IL-1β and IFN-α. CRP indicates tissue damage, promotes inflammation and clearance of apoptotic cells, and presents a potential therapeutic target [21] although with a risk of serious side effects [22]. Chronic macrolide therapy in CF [23] reduces APE perhaps by reducing IL-1β levels [24]. Our finding suggests that increased IL-1β is associated with increased inflammation during an APE. IFN-α levels are acutely elevated with viral infection [25], possibly reflecting viral triggering of an APE [26]. Changes in IFN-α levels alone were greater in the transition from stable-to-APE than APE-to-stable suggesting APE-associated increases in persistent inflammation and permanent tissue damage. However, patients with higher IFN-α levels had fewer prior APE (Table 5). Each additional unit of IFN-α (log scale pg/ml) was associated with 64% fewer prior APE (95% CI, 26-82%). Without a definite association with future APE (Tables 5 and 6), further investigation is needed to confirm a protective role.

Our study of associations between biomarkers and concurrent clinical variables focused on three predictive of survival in CF: FEV_1_%, APE and weight [27]. We found strong independent associations of IL-17A and HMGB-1 with FEV_1_%; IL-17A with weight-for-age *z*-score; and IFN-α and HMGB-1 with number of APE in the year prior to sample collection (Table 5).

IL-17 is elevated in lower CF airways, co-localizes immunohistochemically with neutrophils and mononuclear cells, and increases production of pro-neutrophilic mediators including GM-CSF and IL-8 [28]. Our IL-17A findings are consistent [28]. However, we did not find an association with future clinical outcomes, thus further investigation is needed to find stronger evidence of a causal role in CF airway inflammation.

REFERENCES

1. Kelly MM, Leigh R, Carruthers S, Horsewood P, Gleich GJ, et al. (2001) Increased detection of interleukin-5 in sputum by addition of protease inhibitors. Eur. Respir. J 18: 685–691.

2. Kelly MM, Keatings V, Leigh R, Peterson C, Shute J, et al. (2002) Analysis of fluid-phase mediators. Eur Respir J Suppl 37: 24s–39s.

3. Bonfield TL, Panuska JR, Konstan MW, Hilliard KA, Hilliard JB, et al. (1995) Inflammatory cytokines in cystic fibrosis lungs. Am J Respir Crit Care Med 152: 2111–8.

4. Sagel SD, Chmiel JF, Konstan MW (2007) Sputum biomarkers of inflammation in cystic fibrosis lung disease. Proc Am Thorac Soc 4: 406–417. doi:10.1513/pats.200703-044BR

5. Moser C, Jensen PØ, Pressler T, Frederiksen B, Lanng S, et al. (2005) Serum concentrations of GM-CSF and G-CSF correlate with the Th1/Th2 cytokine response in cystic fibrosis patients with chronic Pseudomonas aeruginosa lung infection. APMIS 113: 400–409. doi:10.1111/j.1600-0463.2005.apm_142.x

6. Hartl D, Griese M, Kappler M, Zissel G, Reinhardt D, et al. (2006) Pulmonary T(H)2 response in Pseudomonas aeruginosa-infected patients with cystic fibrosis. J. Allergy Clin. Immunol 117: 204–211. doi:10.1016/j.jaci.2005.09.023

7. Drumm ML, Konstan MW, Schluchter MD, Handler A, Pace R, et al. (2005) Genetic modifiers of lung disease in cystic fibrosis. N Engl J Med 353: 1443–1453. doi:10.1056/NEJMoa051469

8. Bruscia EM, Zhang P-X, Ferreira E, Caputo C, Emerson JW, et al. (2009) Macrophages directly contribute to the exaggerated inflammatory response in cystic fibrosis transmembrane conductance regulator-/- mice. Am. J. Respir. Cell Mol. Biol 40: 295–304. doi:10.1165/rcmb.2008-0170OC

9. Hubeau C, Le Naour R, Abély M, Hinnrasky J, Guenounou M, et al. (2004) Dysregulation of IL-2 and IL-8 production in circulating T lymphocytes from young cystic fibrosis patients. Clin. Exp. Immunol 135: 528–534.

10. Yang Y, Trinchieri G, Wilson JM (1995) Recombinant IL-12 prevents formation of blocking IgA antibodies to recombinant adenovirus and allows repeated gene therapy to mouse lung. Nat. Med 1: 890–893.

11. McAllister F, Henry A, Kreindler JL, Dubin PJ, Ulrich L, et al. (2005) Role of IL-17A, IL-17F, and the IL-17 receptor in regulating growth-related oncogene-alpha and granulocyte colony-stimulating factor in bronchial epithelium: implications for airway inflammation in cystic fibrosis. J. Immunol 175: 404–412.

12. Best LG, Ferrell RE, Decroo S, North KE, Maccluer JW, et al. (2009) Genetic and other factors determining mannose-binding lectin levels in American Indians: the Strong Heart Study. BMC Med. Genet 10: 5. doi:10.1186/1471-2350-10-5

13. Davé SH, Tilstra JS, Matsuoka K, Li F, DeMarco RA, et al. (2009) Ethyl pyruvate decreases HMGB1 release and ameliorates murine colitis. Journal of Leukocyte Biology 86: 633 –643. doi:10.1189/jlb.1008662

14. Zar JH (1998) Biostatistical Analysis. 4th ed. Prentice Hall.

15. R Development Core Team (2010) R: A language and environment for statistical computing. Vienna, Austria: R Foundation for Statistical Computing. Available: http://www.R-project.org.

16. Cox DR (1972) Regression models and life-tables. J R Stat Soc B Met 34: 187–220. doi:10.2307/2985181

17. Cox DR, Oakes D (1984) Analysis of survival data. CRC Press.

18. Sing T, Sander O, Beerenwinkel N, Lengauer T (2005) ROCR: visualizing classifier performance in R. Bioinformatics 21: 3940–3941. doi:10.1093/bioinformatics/bti623

19. Davison AC, Hinkley DV (1997) Bootstrap Methods and Their Application. 1st ed. Cambridge University Press.

20. Sagel SD, Sontag MK, Wagener JS, Kapsner RK, Osberg I, et al. (2002) Induced sputum inflammatory measures correlate with lung function in children with cystic fibrosis. J Pediatr 141: 811–7.

21. Pepys MB, Hirschfield GM (2003) C-reactive protein: A critical update. J. Clin. Invest. 111: 1805–1812. doi:10.1172/JCI18921

22. Sjöwall C, Zickert A, Skogh T, Wetterö J, Gunnarsson I (2009) Serum levels of autoantibodies against C-reactive protein correlate with renal disease activity and response to therapy in lupus nephritis. Arthritis Res. Ther 11: R188. doi:10.1186/ar2880

23. Saiman L, Marshall BC, Mayer-Hamblett N, Burns JL, Quittner AL, et al. (2003) Azithromycin in patients with cystic fibrosis chronically infected with Pseudomonas aeruginosa: a randomized controlled trial. JAMA 290: 1749–1756. doi:10.1001/jama.290.13.1749

24. Bosnar M, Bošnjak B, Čužić S, Hrvačić B, Marjanović N, et al. (2009) Azithromycin and clarithromycin inhibit lipopolysaccharide-induced murine pulmonary neutrophilia mainly through effects on macrophage-derived Granulocyte-Macrophage Colony-Stimulating Factor and Interleukin-1β. J Pharmacol Exp Ther 331: 104 –113. doi:10.1124/jpet.109.155838

25. Isaacs A, Lindenmann J (1957) Virus interference. I. The interferon. P Roy Soc Lond B Bio 147: 258–267.

26. Wang EE, Prober CG, Manson B, Corey M, Levison H (1984) Association of respiratory viral infections with pulmonary deterioration in patients with cystic fibrosis. N. Engl. J. Med 311: 1653–1658. doi:10.1056/NEJM198412273112602

27. Liou TG, Adler FR, Fitzsimmons SC, Cahill BC, Hibbs JR, et al. (2001) Predictive 5-year survivorship model of cystic fibrosis. Am J Epidemiol 153: 345–52.

28. Brodlie M, McKean MC, Johnson GE, Anderson AE, Hilkens CMU, et al. (2010) Raised interleukin-17 is immuno-localised to neutrophils in cystic fibrosis lung disease. Eur Respir J 37: 1378–1385. doi:10.1183/09031936.00067110
